# Maternal hepatitis B virus carrier status and pregnancy outcomes: a prospective cohort study

**DOI:** 10.1186/s12884-016-0884-1

**Published:** 2016-04-26

**Authors:** Ai-Ming Cui, Xiao-Yan Cheng, Jian-Guo Shao, Hai-Bo Li, Xu-Lin Wang, Yi Shen, Li-Jing Mao, Sheng Zhang, Hai-Yun Liu, Lei Zhang, Gang Qin

**Affiliations:** Department of Obstetrics, The Obstetrics & Gynecology Hospital of Nantong University, Nantong, Jiangsu China; Center for Liver Diseases, Nantong Third People’s Hospital, Nantong University, Nantong, Jiangsu China; Department of Clinical Laboratory, The Obstetrics & Gynecology Hospital of Nantong University, Nantong, Jiangsu China; Department of Epidemiology and Medical Statistics, School of Public Health, Nantong University, 9 Se-Yuan Road, Nantong, Jiangsu 226000 China; Faculty of Medicine, Nursing and Health Science, Monash University, Melbourne, VIC Australia

**Keywords:** Pregnancy, Hepatitis B virus infection, Miscarriage

## Abstract

**Background:**

Infection with hepatitis B virus (HBV) in pregnant women may be a threat for both mothers and fetuses. This study was performed to explore the impact of maternal HBV carrier status on pregnancy outcomes.

**Methods:**

We conducted a prospective cohort study at the Obstetrics & Gynecology Hospital of Nantong University between January 1, 2012 and September 30, 2015. A consecutive sample of 21,004 pregnant women, 513 asymptomatic HBV carriers and 20,491 non-HBV controls, was included in this study. The main outcomes of interest were selected pregnancy outcomes including miscarriage, stillbirth, preterm birth (PTB), gestational diabetes (GDM), intrahepatic cholestasis of pregnancy (ICP), preterm premature rupture of the membrane (PPROM), low birth weight (LBW), small for gestational age (SGA) and Apgar scores. The incidence of adverse pregnancy outcomes between asymptomatic HBV carriers and non-HBV controls were compared using the chi-square test and logistic regression. *P* values were two sided, and *P* <0.05 was considered to indicate statistical significance.

**Results:**

The incidences of stillbirth, PTB, GDM, ICP, PPROM, LBW, and SGA were similar between the HBV carrier and non-HBV groups. The proportion of miscarriage was significantly higher among the HBV carriers than the controls (9.36 % vs 5.70 %; *P* <0.001). After using multivariate modelling to adjust for possible socio-demographical variables and obstetric complications, women with HBV carrier status were still more likely to have miscarriage (adjusted OR 1.71, 95 % CI 1.23–2.38). In addition, the incidences of other maternal and neonatal outcomes were similar between the two groups.

**Conclusion:**

Maternal HBV carrier status may be an independent risk factor for miscarriage and careful surveillance is warranted.

## Background

Hepatitis B virus (HBV) infection is one of the most common health problems worldwide. The prevalence of HBV infection among women of childbearing age may be as high as 2–8 % in China [1, 2], whereas in the USA it is only 0.4 % [3]. Most pregnant women with HBV infection are chronic carriers, indicated by positive serum hepatitis B surface antigen (HBsAg) status. HBsAg expression has also been found in cells of the ovarian follicle or placental capillary endothelium [4]. Intrauterine infection and vertical transmission of HBV is a fundamental reason why there are so many chronic HBV carriers in China. The overall estimated rates of immunoprophylaxis failure for infants with HBsAg-positive and HBeAg-positive mothers were 4.87 and 9.66 % respectively [5].

Although HBV carrier status is relatively common among pregnant women, especially in highly endemic countries such as China, there is a paucity of data regarding the impact of maternal HBV infection on the risk for adverse pregnancy outcomes. The limited studies on this issue have always yielded conflicting results [6–15].

Due to this dearth of information, we undertook this hospital-based prospective cohort study, which seeks to examine the association of HBV carrier status with pregnancy outcomes.

## Methods

### Study design and participant population

We conducted a prospective cohort study at the Obstetrics & Gynecology Hospital of Nantong University, China between January 1, 2012 and September 30, 2015. All pregnant women who visited the tertiary teaching hospital were screened and recruited through 2012 to 2014. Data on maternal characteristics (age, education, medical history) were taken from questionnaires completed by women after their first antenatal visit. Body mass index (BMI) was classified according to the WHO classification: underweight (<18.5 kg/m^2^), normal weight (18.5–24.9 kg/m^2^), overweight (25–29.9 kg/m^2^) or obesity (≥30 kg/m^2^) [16]. As part of standard prenatal care at our hospital, all women are screened in the first trimester of pregnancy for hepatitis B surface antigen (HBsAg), hepatitis B e antigen (HBeAg), IgG antibodies against HCV and HIV, syphilis tests with the treponema pallidum particle agglutination assay (TP-PA) and RPR, specific IgM antibodies against toxoplasma, rubella virus, CMV and HSV-1/2.

All enrolled participants fulfilled the following criteria: (i) normal alanine transaminase (ALT) at study entry; (ii) no evidence of hepatitis C virus (HCV) infection (anti-HCV negative), (iii) absence of human immunodeficiency virus (HIV) infection (anti-HIV negative) and active syphilis infection (rapid plasma reagin test/RPR negative); (iv) absence of IgM antibodies against toxoplasma (TOX), rubella virus (RV), cytomegalovirus (CMV), herpes simplex virus (HSV-1/2); (v) exclusion of other liver diseases such as alcoholic liver diseases (ALDs), nonalcoholic fatty liver diseases (NAFLDs) or autoimmune liver diseases (AILDs); (vi) absence of pre-existing chronic diseases such as diabetes mellitus, hypertension, or heart diseases. HBV carriers were defined as: HBsAg positivity >6 months confirmed by previous medical history, and persistently normal levels of ALT before and at study entry. In addition, HBV carriers were excluded if they had received antiviral treatment within the previous year.

**Fig. 1:**
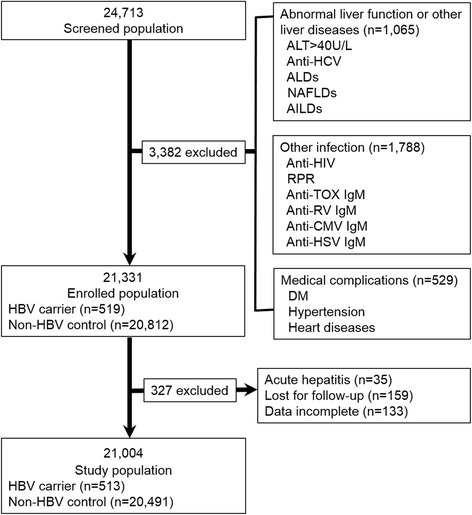
Flow chart for study population. HBV, hepatitis B virus; ALT, alanine transaminase; HCV, hepatitis C virus; HIV human immunodeficiency virus; RPR, rapid plasma reagin test; TOX, toxoplasma; RV, rubella virus; CMV, cytomegalovirus, HSV, herpes simplex virus; ALD, alcoholic liver disease; NAFLD, nonalcoholic fatty liver diseases; AILD, autoimmune liver disease; DM, diabetes mellitus

A total of 24,713 pregnant women were reviewed and screened in their first trimester of pregnancy at the Obstetrics & Gynecology Hospital of Nantong University, China. Of these, 3382 were excluded: due to abnormal ALT or other liver diseases (HCV infection, ALDs, NAFLDs, or AILDs) in 1065 subjects; due to other infection (HIV infection, active syphilis infection, positivity of anti-TOX IgM, anti-RV IgM, anti-CMV IgM or anti-HSV IgM) in 1788 subjects; due to concurrent medical complications (DM, hypertension or heart diseases) in 529 subjects. Thereafter, 21,331 pregnant women were enrolled in this study, including 519 asymptomatic HBV carriers and 20,812 non-HBV controls. Most of the enrolled subjects received at least three health examinations and were followed-up until delivery or miscarriage. 327 subjects were excluded because of the following reasons: acute hepatitis in 35 subjects (hepatitis E in 27 subjects and hepatitis A in eight subjects); lost to follow-up in 159 subjects; incomplete data in 133 subjects. Finally, 21,004 subjects, with 513 asymptomatic HBV carriers (HBeAg positive in 159 subjects) and 20,491 non-HBV controls, were included to evaluate the effect of chronic HBV carrier status on the pregnancy outcomes (Fig. 1).

The study protocol was approved by the institutional ethics committee of the Obstetrics & Gynecology Hospital of Nantong University and conducted in accord with the guidelines of the Declaration of Helsinki. Written informed consent was obtained from each patient.

### Pregnancy outcomes

Primary pregnancy outcomes were: miscarriage (spontaneous abortion), preterm (<37w), or stillbirth (after 20 completed gestational weeks). Other maternal outcomes included preeclampsia, gestational diabetes (GDM), placenta previa, placental abruption, and preterm premature rupture of the membranes (PPROM). Neonatal outcomes for singleton pregnancy included low birth weight (LBW, <2,500 g) or macrosomia (≥4,000 g), small for gestational age (SGA; defined as a birth weight below the 10th percentile for each gestational age using sex-specific criteria) [17, 18] and Apgar score (<7 at 5 min). The rate of cesarean section was not assessed because China has nearly 50 % caesarean delivery rate due to “social influence” rather than medical or obstetric indication [19].

### Statistical analysis

According to a recent survey conducted in China, the prevalence of HBsAg among pregnant women was 6.1 % [1]. With the assumption that the ratio of HBV carriers to non-HBV subjects was 1:20, the baseline risk for adverse pregnancy outcome was 5 % and there might be 2-fold increase of risk with a power of 80 %, the minimum sample size of approximately 4200 participants including 200 HBV carriers were required.

The continuous data were expressed as mean ± standard deviation (SD) and categorical data as number or percent. Comparison of continuous variables was done by student *t* test. For categorical variables the chi-square or Fisher’s exact test were used. Selected maternal and neonatal outcomes in all pregnancies or singleton pregnancies were calculated per woman and/or per infant when appropriate. In this analysis, miscarriage was taken as the dependent variable. Demographic, education, medical history, and laboratory factors were taken as independent variables. Risk factors found significant on univariate analysis were entered into multivariate logistic regression model. All statistical tests were 2-tailed, and a significance level (*P*) of 0.05 was used. The statistical analyses were performed with Stata version 13 (StataCorp, USA).

## Results

### Participant characteristics

The demographic and clinical data of 21,004 study subjects are shown in Table 1. The mean age (SD) of the asymptomatic HBV carrier group was slightly but significantly older than that of the control group (27.59 ± 4.02 vs. 27.03 ± 4.19 years, *P* <0.01). But no significant difference was found between the two groups in terms of the proportion of older pregnant women (aged 35 years or more). HBV carriers also had significantly higher serum ALT levels than controls (24.75 ± 7.22 vs. 22.34 ± 6.49 U/L, *P* < 0.001). Moreover, there was no significant difference in terms of height, pre-maternal BMI, education level, parity, history of miscarriage or preterm birth, plurality, and in vitro fertilization (IVF) between the HBV carriers and the controls.

**Table 1:** Baseline characteristics of the study population^a^

Characteristic	HBV carriers (*n* = 513)	Controls (*n* = 20491)	*P*
Maternal age (y)	27.59 ± 4.02	27.03 ± 4.19	0.003
≤19	2	239	0.234
20–34	476	18,974	
≥35 (older pregnancy)	35	12,78	
Height (cm)	156.23 ± 8.43	155.81 ± 7.32	0.201
Pre-pregnancy BMI	22.34 ± 5.86	22.02 ± 5.69	0.209
<18.5	35	1,751	0.396
18.5–24.9	408	16,137	
25–29.9	67	2,536	
≥30	3	67	
Education			
Primary/under	15	892	0.288
Middle/High school	171	6,785	
College/above	327	12,814	
Parity			
0	373	15,524	0.258
1	126	4,519	
≥2	14	448	
Previous abortion			
0	456	18,607	0.385
1	42	1,329	
≥2	15	555	
Previous preterm birth			0.907
0	504	20,168	
1	9	320	
≥2	0	3	
Plurality			
1	497	20,001	0.288
≥2	16	490	
IVF			
No	509	20,344	0.869
Yes	4	147	
ALT (U/L)	24.75 ± 7.22	22.34 ± 6.49	<0.001

*Abbreviations*: *BMI* body mass index, *IVF* in vitro fertilization

^a^Values are reported as mean ± SD, compared with *t* test, or number, compared with *χ* ^2^ test, unless otherwise indicated

During the follow-up, 20885 patients had persistently normal levels of serum ALT, while 119 (0.57 %) patients experienced at least one episode of ALT > the upper limit of normal (ULN, 40U/L). Among them, 6 HBV carriers and 112 non-HBV controls showed a mild ALT increase (≤2ULN), while only 1 non-HBV subject showed a moderate ALT increase (>2ULN). All the ALT elevation was transient and usually recovered to normal levels within 3 months.

### Pregnancy outcomes

The mean (SD) of completed weeks’ gestation was significantly shorter in HBV carriers than in the non-HBV controls (35.79 ± 8.29 vs. 36.95 ± 6.76; *P* <0.001). The proportion of miscarriage was significantly higher among the HBV carriers than the controls (9.36 % vs 5.70 %; *P* <0.001). Meanwhile, there was no significant difference between the two groups for most of the pregnancy outcomes such as preterm birth, stillbirth, GDM, ICP, placenta previa or PPROM (Table 2).

**Table 2:** Comparison of the pregnancy outcomes between asymptomatic HBV carriers and non-HBV controls^a^

Outcome	HBV carriers (*n* = 513)	Controls (*n* = 20,491)	*P*
Completed weeks’ gestation	35.79 ± 8.29	36.95 ± 6.76	<0.001
≤12 wks	36	822	0.002
12 1/7 to 28 wks	13	390	
28 1/7 to 40 wks	369	14,755	
>40 wks	95	4,524	
Preterm birth	49 (9.55 %)	1,718 (8.38 %)	0.347
Stillbirth	0	18 (0.09 %)	0.502
Miscarriage	48 (9.36 %)	1,167 (5.70 %)	<0.001
Preeclampsia	4 (0.78 %)	216 (1.05 %)	0.600
GDM	6 (1.17 %)	232 (1.13 %)	0.937
ICP	12 (2.34 %)	331 (1.62 %)	0.201
Placenta previa	4 (0.78 %)	216 (1.05 %)	0.547
PPROM	23 (4.48 %)	845 (4.12 %)	0.686

*Abbreviations*: *GDM* Gestational diabetes, *ICP* intrahepatic cholestasis of pregnancy, *PPROM* preterm premature rupture of the membrane

^a^Values are reported as mean ± SD, compared with *t* test, or number (percentage), compared with *χ* ^2^ test, unless otherwise indicated

In singleton pregnancy, the rate of miscarriage was also significantly higher in HBV carrier group than in the control group (9.66 % vs. 5.81 %, *P* <0.001). Preterm birth occurred in 36/497 (7.24 %) and 1462/20,001 (7.31 %) of pregnancies in HBV carrier and control group respectively (*P* >0.05). The mean (SD) birth weight was 3311.26 (510.34) g and 3330 (509.08) g in the two groups respectively (*P* >0.05). Comparable neonatal adverse outcomes occurred in the two groups (e.g., <1,500 g: 0.80 % vs. 0.54 %; <2,500 g: 5.03 % vs. 4.52 %; SGA: 4.43 % vs. 5.16 %; respectively). There were no significant differences in incidences of perinatal mortality or Apgar score <7 at 5 min (Table 3).

**Table 3:** Neonatal outcomes in singleton pregnancy^a^

Outcome	HBV carriers (*n* = 497)	Controls (*n* = 20,001)	*P*
Full-term birth	413 (83.10 %)	17,364 (86.82 %)	0.016
Preterm birth	36 (7.24 %)	1462 (7.31 %)	0.955
Stillbirth	0	12 (0.06 %)	0.585
Miscarriage	48 (9.66 %)	1163 (5.81 %)	<0.001
Weight of neonates (g)	3311.26 ± 510.34	3330 ± 509.08	0.436
Unknown	0	9	
<1500 g	4	108	0.585
1500–2500 g	21	797	
2500–3999 g	392	17,489	
≥4000 g	32	1,598	
LBW			
Unknown	0	9	
No	472	19,087	0.594
Yes	25	905	
SGA <10 centile			
Unknown	0	9	0.461
No	475	18,959	
Yes	22	1,033	
Apgar at 5 min	9.90 ± 0.53	9.87 ± 0.69	0.336
≥7	488	19,744	0.292
<7	9	257	

*Abbreviations*: *LBW* low birth weight, *SGA* small for gestational age

^a^Values are reported as mean ± SD, compared with *t* test, or number (percentage), compared with *χ* ^2^ test, unless otherwise indicated

### Risk factors associated with miscarriage

Table 4 presents crude and adjusted odds ratios (ORs) and 95 % confidence intervals (CIs) of miscarriage associated with demographic, physical, reproductive and laboratory characteristics. When single factors were analyzed for the risk of miscarriage, five factors had been identified: (i) history of miscarriage with crude OR of 7.95 (95 % CI 6.85–9.22) for once and crude OR of 18.89 (95 % CI 15.72–22.71) for twice or more respectively; (ii) in vitro fertilization (crude OR 2.37, 95 % CI 1.46–3.84); (iii) older age (crude OR 2.22, 95 % CI 1.85–2.66); (iv) HBV carrier status (crude OR 1.71, 95 % CI 1.26–2.31); and (v) nulliparity (crude OR 1.48, 95 % CI 1.31–1.69). We did not find the association of education level, pre-maternal BMI with miscarriage risk. Furthermore, we examined the covariates associated with miscarriage using multivariable logistic regression. Factors independently associated with miscarriage were: prior abortion (adjusted OR 9.33 for once, adjusted OR 23.36 for twice or more), nulliparity (adjusted OR 1.80), IVF (adjusted OR 1.78), and HBV carrier status (adjusted OR 1.71).

**Table 4:** Risk factors associated with miscarriage

	Cases/Exposed	Crude OR (95 % CI)	Adjusted OR (95 % CI)
Age			
<35	1067/19691	1	1
≥35	148/1313	2.22 (1.85–2.66)	1.17 (0.94–1.45)
Nulliparity			
No	830/15897	1	1
Yes	385/5107	1.48 (1.31–1.69)	1.80 (1.54–2.11)
Education			
High school/under	497/8063	1	
College/above	718/12941	0.83 (0.67–1.19)	
Pre-maternal BMI			
<25	1049/18331	1	
≥25	166/2673	1.09 (0.97–1.42)	
Previous abortion			
0	673/19063	1	1
1	309/1371	7.95 (6.85–9.22)	9.33 (7.97–10.92)
≥2	233/570	18.89 (15.72–22.71)	23.36 (19.10–28.57)
HBV carrier			
No	1167/20491	1	1
Yes	48/513	1.71 (1.26–2.31)	1.71 (1.23–2.38)
IVF			
No	1196/20853	1	1
Yes	19/151	2.37 (1.46–3.84)	1.78 (1.03–3.05)

*Abbreviations*: *BMI* body mass index, *IVF* in vitro fertilization

## Discussion

Our prospective cohort study shows that the incidence of miscarriage in pregnant women with chronic HBV infection is significantly higher than that in non-HBV controls. The association remain unchanged after adjustment for other possible maternal confounders such as age, parity and history of abortion. This result is surprising. We for the first time report that HBV carrier status entails a considerable increased risk of miscarriage.

The relationship between viral infections and miscarriage has not been well-understood. On the one hand, several viruses such as CMV, HSV-1/2, human parvovirus B19 (HPV B19), adenovirus, Coxsackie B virus, have been indicated to be causative agents of miscarriage [20, 21]. On the other hand, the causal relationship of other viruses with miscarriage has not been established for some reasons. First, viral infection of the pregnant women may not always represent fetal infection because of the placental barrier. Second, placental viral infection indicates risk of vertical transmission, but not always transmission to, or disease of the fetus. Last, the intrauterine viral infection does not always cause morbidity or mortality at the early stage of the fetus. For example, the rate of miscarriage following acute varicella did not exceed the rate of miscarriage in pregnant women without chickenpox [22].

The question whether HBV infection in the mothers entails an increased risk for adverse pregnancy outcomes was raised in the 1960s [23], and has been followed by several studies with contradictory and confusing results. For the case–control studies [8–10], the selection bias is a big concern. For the retrospective cohort studies [11, 13–15], information for early pregnancy is incomplete. For the prospective cohort studies [7, 12], small sample sizes (with 1576 and 1826 participants respectively) are their major limitation. To the best of our knowledge, this is the first prospective single-center study on HBV carriers during pregnancy to date. Our finding implies that chronic HBV carrier status is likely to affect placental function rather than preterm labor.

The study population had a high level of homogeneity in that nearly all participants were ethnic Han from an eastern region of China and all were asymptomatic. Although the prevalence of HBsAg (2.5 %) among pregnant women in this study was much lower than that (6.1 %) in another survey conducted in a southwest region of China in 2010 [1], its epidemiologic power is still strong. In such a sample of more than 500 women who were exposed to chronic HBV infection in pregnancy with a ratio of 1:40 to the controls and the assumption of a baseline miscarriage risk of 5 %, the detection of a 1.7-fold increase in the rate of miscarriage (with 95 % CI), yielded a power of 90 %.

It should be noted that in this study asymptomatic HBV carriers had significantly higher serum ALT levels than non-HBV controls, suggesting that even in clinically asymptomatic infection, HBV may still produce adverse effects on the liver. Their longer-term effects on the liver as well as metabolic profiles await further examinations. Asymptomatic HBV carriers can be categorized into two clinical HBV phases: immune tolerance (IT) and inactive carrier (IC) [24]. IT patients had positive serum HBeAg and repetitive normal ALT values (<40 IU/L) for at least 1 year. Patients with typical IT form (very high viral load HBV-DNA ≥2 × 10^6^ IU/mL) have very limited liver injury, while patients with atypical form (HBV-DNA <2 × 10^6^ IU/mL) have a potential risk of more severe histological lesions. IC patients had repetitive normal ALT values (<40 IU/L) for at least 1 year, negative HBeAg and positive HBeAb. Patients with typical IC form have low viral load (HBV-DNA <2,000 IU/mL) and minimal histological lesions, while patients with atypical form had higher HBV-DNA (≥2,000 IU/mL) remain problematic. Therefore, the absence of symptoms is not enough to accurately separate patients with significant progressive liver injury from inactive HBsAg carriers [25].

Our study has shown that the majority of pregnant women maintain persistently normal levels of serum ALT through the pregnancy. Elevation of ALT was usually within 1–2 × ULN, and decrease to normal levels within three months. The incidence of abnormal ALT is 0.57 % through the study. This rate seems significantly lower than the estimated incidence rate of 3 % for abnormal liver functions as previously reported in other population studies [26]. A possible explanation may be the inclusion criteria of this study, i.e. no pre-existing chronic liver diseases, no concurrent medical diseases, treatment-naive HBV carrier status, and normal ALT level at study entry.

The outcome of a woman’s previous pregnancy has profound consequences for her subsequent pregnancies. We found that the most important predictive factor for miscarriage is a history of spontaneous or induced abortion, in accordance with previous studies [27, 28]. The etiology of recurrent pregnancy loss is still unclear [29]. While induced abortion may increase the risks for both a subsequent preterm delivery and mood disorders substantial, the association of previous induced abortion with subsequent miscarriage remains controversial [30].

Admittedly, our study has several limitations. First of all, we were only able to locate very few data regarding viral load in these HBV carriers. Thus, we were unable to conclude any significant correlation between higher viral loads at the time of pregnancy and adverse outcomes. Second, some potential confounders which might affect pregnancy outcomes have not been included. For example, cigarette, alcohol, and caffeine consumption may be risk factors for miscarriage. Actually, in Chinese culture, women are discouraged from drinking alcohol and from smoking cigarette. Most people drink tea over coffee in China as well. Another limitation is single-center nature of this study. Indeed, the Obstetrics & Gynecology Hospital of Nantong University is the sole special hospital for the treatment of women illnesses in Nantong city, eastern China, and serves more than half of obstetric population in this region.

## Conclusion

This study reflects the clinical reality of pregnant women and indicates that HBV carrier status confers a substantial increased risk for miscarriage. Careful surveillance as high-risk pregnancy is warranted. Further studies are needed to assess the importance of factors such as disease activity, viral characteristics, and other clinical features of the disease.

### Ethics

The study protocol was approved by the institutional ethics committee of the Obstetrics & Gynecology Hospital of Nantong University and conducted in accord with the guidelines of the Declaration of Helsinki. Written informed consent was obtained from each patient.

### Availability of data and materials

The datasets supporting the conclusions of this article is included within the manuscript.

## Abbreviations

AILD: autoimmune liver disease
ALD: alcoholic liver disease
ALT: alanine transaminase
BMI: body mass index
CI: confidence interval
CMV: cytomegalovirus
DM: diabetes mellitus
GDM: gestational diabetes
HBeAg: hepatitis B e antigen
HBsAg: hepatitis B surface antigen
HBV: Chronic hepatitis B virus
HCV: hepatitis C virus
HIV: human immunodeficiency virus
HPV: human parvovirus
HSV: herpes simplex virus
IVF: in vitro fertilization
LBW: low birth weight
NAFLD: nonalcoholic fatty liver diseases
OR: odds ratio
PPROM: preterm premature rupture of the membranes
RPR: rapid plasma reagin test
RV: rubella virus
SD: standard deviation
SGA: small for gestational age
TOX: toxoplasma
ULN: upper limit of normal
VZV: varicella-zoster virus
